# Larval nutrition influences adult fat stores and starvation resistance in *Drosophila*

**DOI:** 10.1371/journal.pone.0247175

**Published:** 2021-02-19

**Authors:** Niyas Rehman, Jishy Varghese

**Affiliations:** School of Biology, Indian Institute of Science Education and Research (IISER TVM), Thiruvananthapuram, Kerala, India; Inha University, REPUBLIC OF KOREA

## Abstract

Insulin plays a major role in connecting nutrient availability to energy homeostasis by regulating metabolic pathways. Defects in insulin signalling is the primary cause for diabetes, obesity and various metabolic disorders. Nutritional status during growth and developmental stages play a crucial role in determining adult size, fecundity and ageing. However, the association between developmental nutrition and adult metabolic disorders has not been fully explored. Here, we address the effects of nutrient status during the larval growth phase on adult metabolism in *Drosophila*. We report that restricted food supply in larvae led to higher fat reserves and starvation resistance in mature adult flies, which we attribute to low insulin signalling. A lesser amount of stored fat was mobilised during early adult stages and during acute starvation, which accounts for the metabolic effects. Furthermore, larval diet influenced the expression of fat mobilisation genes *brummer* and *lipid storage droplet-2* in adult flies, which led to the metabolic phenotypes reported here. Thus, the restricted nutrient environment in developing larvae led to adaptive changes that entrain the adult flies for scarce food availability.

## Introduction

Nutrients are inevitable to meet the energy requirements for the normal development and functioning of all living organisms. During periods of excess supply, nutrients are stored as the energy source for phases of limited food availability and for activities that demand excess energy. Both intrinsic genetic factors and environmental influences cause imbalances in nutrient homeostasis [1–6]. Defects in maintaining nutrient balance are responsible for various metabolic disorders like diabetes and obesity [7]. Maintenance of nutrient homeostasis at specific life stages is crucial for the proper functions performed during that life stage [8–14]. However, deviations in nutrient supply during one life stage may affect functions performed during other stages. Recent reports suggest that proper nutrient balance during developmental stages plays a crucial role in managing biological functions in the adult organism [15–20].

Insulin, a highly conserved peptide hormone, regulates metabolic processes by acting at cellular and systemic levels [21]. In vertebrates, insulin is synthesised by the pancreatic beta islet cells and released into the bloodstream upon glycemic demand [22]. Insulin controls glucose homeostasis by aiding in the uptake of glucose from the circulation into the liver and muscle cells. In addition to its roles in glucose homeostasis, insulin manages lipid metabolism and regulates fat cell mass. There is a consensus that insulin promotes triglyceride synthesis in the mammalian liver and the insect fat body by augmenting the expression and activity of various lipogenic factors [23–32]. Activation of the insulin receptor in response to ligand binding triggers a series of phosphorylation events that act through the insulin signalling cascade consisting of PI3K, Akt/PKB, TOR and S6K kinases [33–35]. Further, activation of AKT inhibits the forkhead transcription factor FOXO, a key regulator of fasting induced lipid mobilisation [36]. A functional counterpart of vertebrate insulin exists in *Drosophila*, which controls glucose and fat metabolism, and ensures energy availability for various biological functions [30, 37, 38]. In *Drosophila*, there are eight insulin-like-peptides (DILPs), one insulin receptor (InR) and an intracellular signalling pathway with components similar to that in vertebrates [39]. DILP2, DILP3 and DILP5 are produced mainly by the insulin-producing cells (IPCs), a subset of the median neurosecretory cells (mNSCs), located in the *pars intercerebralis* region of the brain [40, 41]. Besides, the *Drosophila* fat body, which resembles vertebrate liver and adipocytes in function, stores triglycerides and glycogen, the primary energy stores for normal functioning and survival during low food availability [42, 43]. Insulin signalling regulates the balance between lipid biosynthesis and breakdown. However, the exact mechanisms by which insulin manages fat levels in specific physiological contexts, especially in response to fluctuations in the environment, remains elusive.

Defects in insulin signalling have been reported to result in a plethora of effects in *Drosophila*, which include growth, metabolism, life-span, responses to stress and fecundity [44–47]. Reduced activity of the insulin signalling pathway led to increased lipid accumulation in flies, contrary to what was expected based on mammalian studies. Flies with mutations in the genes that encode the *insulin receptor* (InR) or the insulin receptor substrate *chico* are obese [44, 47]. Ablation of IPCs or downregulation of *dilp* gene expression in the IPCs resulted in a significant increase in stored fat levels [44, 47]. Expression of a FOXO mutant form, which cannot be inhibited by AKT, in the head fat body led to the accumulation of enlarged lipid droplets [48]. However, other studies in *Drosophila* show an opposite trend, as expected from mammalian studies, activation of insulin signalling led to an increase in the number of fat cells and accumulation of stored fat. Also, clones overexpressing *inr* or the catalytic subunit of PI3K (*dp110*) in the larval fat body showed an accumulation of lipid droplets [23, 24]. The reasons for this discrepancy in reports from different groups that use *Drosophila* as a model to elucidate the role of insulin signalling on lipid metabolism is not yet clear.

During acute food shortage animals mobilize stored energy resources accumulated during periods of excessive nutrient supply. Reduced activity of insulin signalling pathway in response to low nutrient levels culminate in mobilisation of stored energy resources. Fasting stimulates the breakdown of triglycerides stored in adipocyte lipid droplets, which meets the energy requirements of the organism despite the low nutrient status. Lipid mobilization during food deprivation is controlled by lipolytic hormones that act via the ß-adrenergic receptor, which subsequently acts through the hormone-sensitive lipase HSL and the scaffold protein perilipin at the lipid droplet surface [49–52]. Adipocyte triglyceride lipase (ATGL), strongly enriched in adipose tissue, acts together with HSL to activate lipolysis in response to fasting [53]. The function of ATGL and perilipin is highly conserved during evolution. In *Drosophila*, starvation triggered fat breakdown is linked to the upregulation of Brummer, an ATGL-like lipase, in the fat body [54]. Fasting induced *bummer* gene expression is dependent on direct regulation by FOXO [55]. Like mammals, lipid mobilization is also negatively regulated by *Drosophila* perilipin Lsd2 (lipid storage droplet-2) [56]. Another factor that aids in fasting-induced lipid utilisation is the *Drosophila* ACS (acetyl-CoA synthetase) encoding gene *pudgy* [57]. Pudgy, a target of the insulin signaling pathway, is strongly upregulated upon fasting under transcriptional control by FOXO. Normal functioning of all the factors mentioned above is crucial for maintaining normal storage fat levels and its mobilisation upon fasting in flies. Defects in the activity of any of these factors influence lipid stores and responses to nutrient deprivation.

Nutrients regulate insulin signalling in *Drosophila* by controlling DILP levels and by acting on insulin signalling pathway at multiple levels (reviewed here). Dietary restriction and low insulin signalling have been shown by previous studies in various organisms to improve their physiological fitness and life span [58–60]. In *Drosophila*, the proper availability of nutrients during larval growth stages is crucial for normal development and attaining proper body size [61]. Reduced insulin signalling in the developmental stages also led to growth phenotypes matching that of nutrient restriction during larval stages [44, 47]. Thus, the size defects caused by reduced nutrient levels in developmental stages are due to low insulin signalling, which also affects relative tissue sizes [62]. Perturbing insulin signalling and changes in diet status in the adult stages led to effects on the adult metabolism and life span [13, 14, 63]. However, the quality and quantity of diet during developmental stages are also believed to have a major influence on the adult life stage [20]. Developmental nutrition has been shown to influence adult physiology at multiple levels; in addition to body size it has been reported to modulate life span and fecundity [18, 64]. However, molecular mechanisms by which dietary status or insulin signalling in growth phases affect adult physiology and metabolism are not well understood.

Recent reports suggest that developmental nutrition may influence the metabolic status of mature stages [65, 66], thus preparing the adult organism to anticipate a similar nutritional environment [67, 68]. However, there are no studies that report that larval diet or larval insulin signalling show an effect on adult nutrient stores, though larval diet influenced ageing and fecundity in adult flies [18, 63]. Though it is expected that reduced larval nutrition may influence the adult flies’ responses to acute depletion of food reserves and aid in their better survival, this has not been reported thus far. The effect of larval nutrition on adult nutrient stores may also be responsible for the effects seen on reproductivity and ageing; this has not been tested yet.

Here, we show that dietary restriction in *Drosophila* larvae led to profound effects on the metabolic status of the mature adult flies. Reduced utilization of lipid stores during early adult stages of flies that emerge from diet restricted larvae contributed to higher fat levels in mature flies. We report that reduced insulin signalling due to low nutrient diet in the larva is responsible for the presence of excess stored fat in the adult flies. Mature adult flies that received restricted larval nutrition showed enhanced resistance to starvation, which is due to a longer retention of stored fat during food deprivation. These flies showed changes in the expression levels of genes that encode for *Drosophila* lipase *brummer* (*bmm*) and perilipin *lipid storage droplet-2* (*lsd2*). Lower levels of *bmm* and higher levels of *lsd2* gene expression together contributed to the fat phenotype and starvation resistance in mature flies that emerged from a diet restricted larvae. Thus, we report that diet during growth and developmental stages influence the metabolic status in adult flies and entrain the organism to resist starvation stress.

## Results

### Nutrient restriction in larvae leads to adult metabolic phenotypes

**Fig 1:**
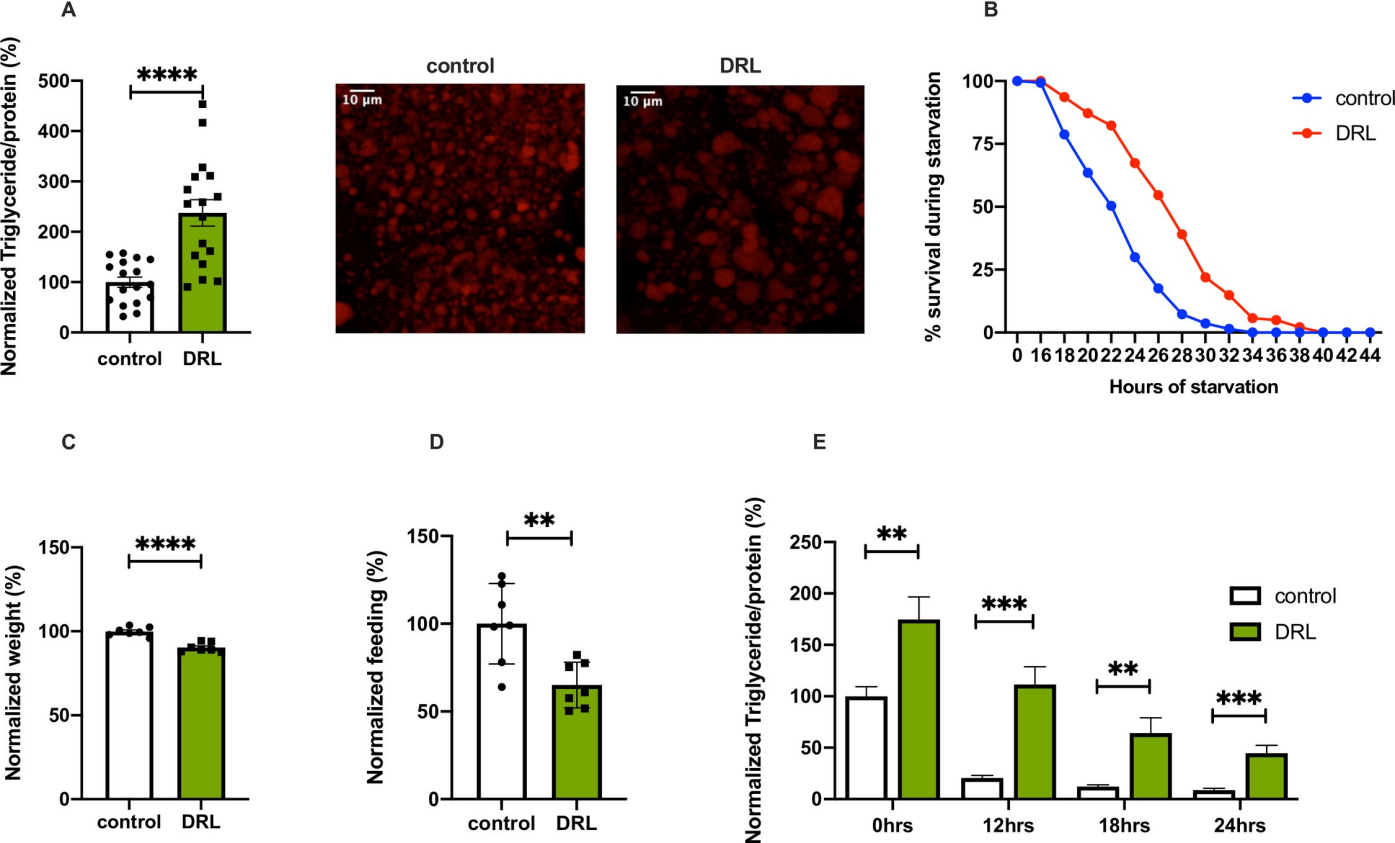
Nutrient restriction in larvae leads to adult metabolic phenotypes. (A) Triglyceride levels are higher in mature flies (5 days old) that emerged from larvae fed with 50% diluted food (DRL) in comparison to control flies, data is shown as % ratio of triglyceride to total protein levels, normalised to 100% in control flies [independent biological replicates = 17, p-value between control and DRL is = 0.0001; Unpaired Students t-test with Welch Correction]. Large lipid droplets are seen in the abdominal fat body of DRL 5 day old flies compared to control upon Nile red staining. (B) Enhanced resistance to starvation in mature DRL flies in comparison to control flies. Data are shown as the percentage of flies of control and DRL, which were alive at various time points of starvation [independent biological replicates = 6, p-value between control and DRL is = 0.0001; Log-rank test, data is presented as mean]. (C) DRL flies were small when compared to control flies, data are shown as the % body weight of flies, normalised to 100% in control flies [independent biological replicates = 7, p-value between control and DRL is = 0.0001; Unpaired Students t-test with Welch Correction]. (D) Hunger induced feeding is reduced in mature DRL adults compared to control flies upon 12 hours of starvation, data is shown as percentage feeding normalised to 100% control flies [independent biological replicates = 7, p-value <0.0044; Unpaired Students t-test with Welch Correction]. (E) Mature DRL flies (5 days old) utilize stored fat slower than control flies during acute starvation, data is represented as the ratio of triglyceride to protein levels in %, normalized to 100% for control flies at 0 hours of starvation, the ratio of triglyceride to protein levels in % is shown for both control and DRL flies after 0, 12, 18 and 24 hours of starvation [independent biological replicates = 12; p-value for 0 hour starvation between control and DRL flies is = 0.0068, p-value for 12 hour starvation between control and DRL flies is = 0.0003; Unpaired Students t-test with Welch Correction, p-value for 18 hour starvation between control and DRL flies is = 0.0011 Mann-Whitney test, and p-value for 24 hour starvation between control and DRL flies is = 0.0007; Unpaired Students t-test with Welch Correction][p-value *<0.05; ** <0.01,*** <0.001, **** <0.0001, data is presented as mean ± SEM].

*Drosophila* larvae were reared on fly food prepared at various dilutions ranging from 25% to 75% of the normal fly media (for details see Materials and methods) to study the effects of larval diet restriction on adult metabolism **(Fig 1, S1 Fig)**. Based on the minimal effects seen on developmental timing and growth **(Fig 1C)**, we decided to use 50% diluted fly media to carry out all further experiments, hereafter referred to as diet restricted larvae (DRL). Further dilution of food did not show any enhancement of the phenotypes observed with DRL **(S1E Fig)**. Male flies that emerged from diet-restricted larvae were aged for five days on normal fly food and various metabolic parameters were analyzed in these mature adult flies. DRL flies showed an increase in triglyceride levels when compared to control flies **(Fig 1A, graph)**; thus, larval food restriction led to enhanced fat stores in mature adult flies. Corresponding to the increase in the levels of stored lipid measurements performed at the level of whole flies, we saw an increase in lipid droplet size in the adult abdominal fat body as well **(Fig 1A, images)**. In addition, we observed that dietary restriction in larval stages led to a significant increase in fat stores in other life stages as well **(S1A and S1B Fig)**. However, in comparison to mature adult flies, these life stages showed a minor impact on fat stores as a result of low larval diet. Further, DRL flies showed enhanced tolerance to complete food withdrawal **(Fig 1B)**. As expected, larvae that were fed on low nutritive food delayed the onset of metamorphosis by ~36 hours and reduced body size **(Fig 1C)**. DRL flies fed less than control flies when reintroduced to food after 12 hours of starvation, implying that due to excess stored fat these flies experience less hunger induced by food withdrawal **(Fig 1D)**. Glycogen levels were slightly reduced (the differences in values were not significant) in mature DRL male flies when compared to control flies; however, circulating levels of glucose was comparable in both conditions **(S1C and S1D Fig)**. Thus, the larval diet affected fat stores and sensitivity to acute nutrient deprivation in mature adult flies. Next, we addressed the potential reasons for the metabolic phenotypes observed in mature adult flies in response to reduced larval nutrition.

### Nutrient restriction in larvae led to changes in fat utilization during early adult stages

**Fig 2:**
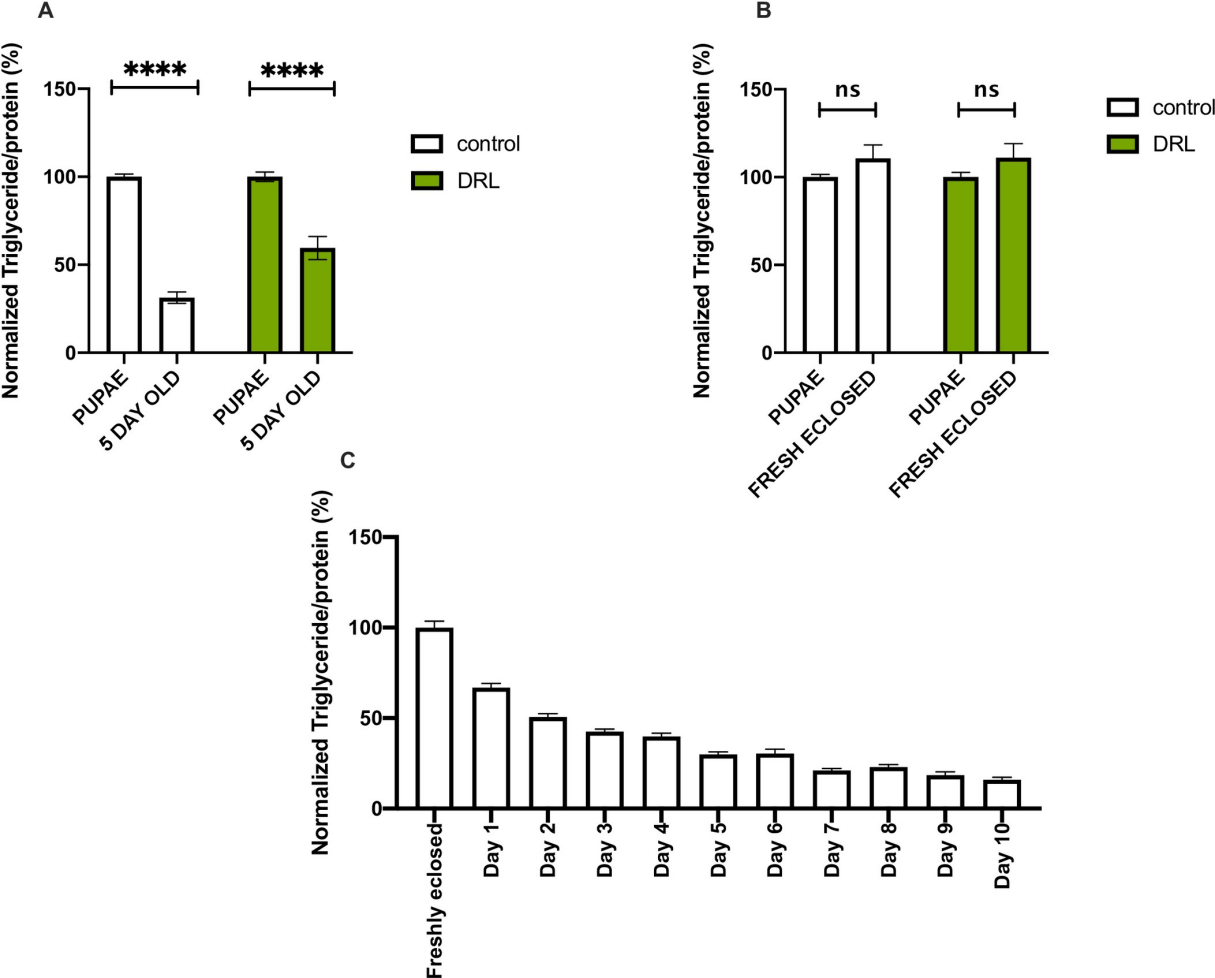
Nutrient restriction in larvae led to changes in fat utilization during early adult stages. (A) Triglyceride levels in early pupae and 5-day old adult flies show that DRL flies utilize less stored fat than control flies. Data is represented as ratio of triglyceride to protein levels in %, normalized to 100% in early pupal stages for both control and DRL [independent biological replicates for control early pupae = 8; independent biological replicates for control 5 day old = 17; independent biological replicates for DRL early pupae = 8; independent biological replicates for DRL 5 day old = 17; p-value between control early pupae and control 5 day flies is = 0.0001; p-value between DRL early pupae and DRL 5 day flies is = 0.0001; p-value between control 5 day flies and DRL 5 day flies is = 0.0001; Unpaired Students t-test with Welch Correction]. (B) There is no significant reduction in triglyceride utilization from pre-pupae to freshly eclosed flies in control and DRL flies, data is shown as percentage ratio of triglyceride to total protein levels, normalised to 100% control flies [control pupae n = 8; control freshly eclosed n = 28; DRL pupae n = 8; Control freshly eclosed n = 28; p-value between control pupae and control freshly eclosed is = 0.1806 and p-value between DRL freshly eclosed and DRL pupae = 0.1998; Unpaired Students t-test with Welch Correction]. (C) There is a reduction in the levels of triglycerides from freshly eclosed to 10 day old adult flies (n = 12).

To address the reasons for excess fat levels observed in mature DRL flies we compared stored fat levels during intervening stages after larvae to 5-day old adults, we also checked fat utilization during the transition from early pupae to freshly eclosed and mature adult stages. During early pupal stages and in freshly eclosed adult flies, low calorie diet led to 25% more fat stores than in control fed conditions **(S1A and S1B Fig)**. Thus, reduced larval feeding affected fat stores during pupal and early adult stages, which could have contributed to the higher fat stores observed in mature DRL adults **(Fig 1A)**. However, a significant reduction in the utilization of fat stores was also observed in DRL animals in comparison to control animals during the transition from early pupal stages to 5-day old flies **(Fig 2A)**. A separate analysis of fat utilization did not show any significant utilization of fat stores during metamorphosis and there were no changes in the utilization of fat in DRL animals in comparison to control fed animals during early pupae to adult transition **(Fig 2B)**. Thus, reduced utilisation of stored fat during early adult stages contributed considerably to the excess fat levels in mature DRL flies.

Further, we measured fat mobilisation during food withdrawal in DRL flies, a change in the ability to mobilise nutrient stores can alter the sensitivity towards acute food deprivation. Fat utilisation was reduced in DRL flies subjected to starvation **(Fig 1E)**; however, the reduced mobilisation of fat in DRL flies did not make them sensitive to starvation stress. At 24 hours of starvation, only ~30% of the control flies survived; in comparison, ~70% of DRL flies were still alive **(Fig 1B)**. The higher initial levels of fat in 5-day old flies and reduced utilisation of the stored fat in response to starvation has led to the retention of 5 fold fat stores after 24 hours of starvation in DRL animals when compared to control animals **(Fig 1E)**. The presence of fat stores even after 24 hours of starvation would have conferred enhanced resistance by enabling the flies to meet their energy demands beyond the stage when control animals start dying. Enhanced energy reserves in adult flies that emerge from diet restricted larvae could also be responsible for the reduced feeding responses observed after food withdrawal for 12 hours **(Fig 1D)**. Next, we investigated the source of excess stored fat in the adult DRL flies.

### Larval fat body retention in mature adult flies that emerge from diet restricted larvae

**Fig 3:**
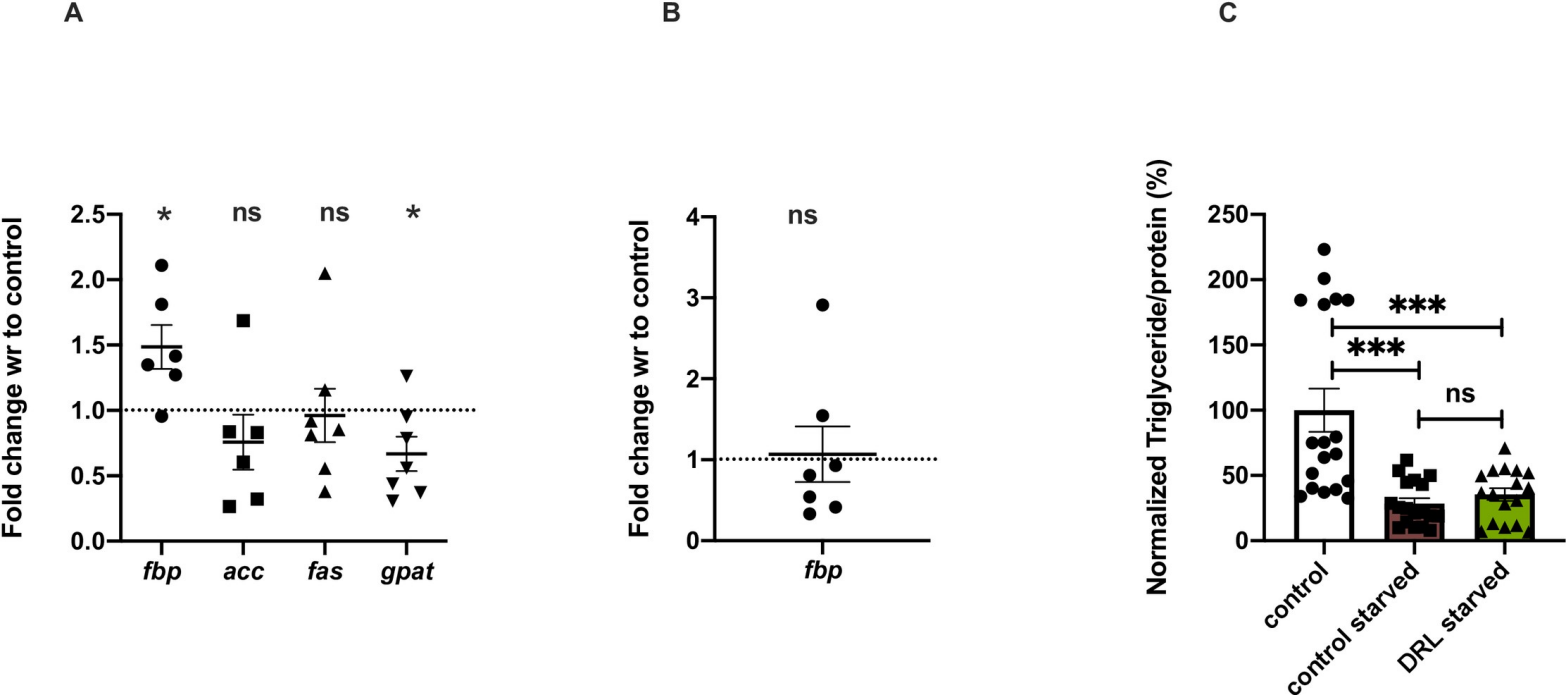
Larval fat body retention in adults results in obese flies. (A) Larval fat body marker *fbp1* is present in 5 day old DRL flies. Shown is *fbp1* mRNA levels in 5-day old control and DRL flies, data are shown as fold change in mRNA levels, values are normalised to control flies and fold change in DRL flies is shown [independent biological replicates = 6; p-value = 0.0340; *acc* [p-value = 0.3010] [independent biological replicates n = 6], *fas* [p-value = 0.8555] and *gpat* [p-value = 0.0450] gene expression in the 5 day old DRL flies. (B) *fbp1* levels in the larvae remain unchanged [independent biological replicates = 6; p-value = 0.8476] in the 3rd DRL larvae, normalised to *rp49* mRNA level is shown [independent biological replicates = 7; Unpaired Students t-test with Welch Correction]. Data are shown as fold change in mRNA levels, values are normalised to control flies and fold change in DRL flies is shown. (C) Triglyceride levels are equal in control and DRL flies after starvation for 48 hours upon eclosion and transferred to normal vials for five days with normal control flies. Data is represented as the ratio of triglyceride to protein levels in %, normalized to 100% in fed control flies, in comparison % of triglyceride to protein ratio is shown for control and DRL flies starved for 48 hours and recovered for five days with normal food [independent biological replicates = 18; p-value between control fed and control starved is = 0.0001, p-value between control fed and DRL starved is = 0.0011 and p-value between control starved and DRL starved is = 0.3346; Mann-Whitney test]. [p-value *<0.05; ** <0.*01*,*** <0.*001*, **** <0.*0001*, *data is presented as mean ± SEM*].

The larval fat body is retained in pupae and adults, which is the key metabolic resource for metamorphosis and early adult functions [69]. Freshly eclosed flies contain a large reserve of fat, including larval fat, which is consumed during early adult stages in males (Aguila et al, 2007). We saw that about 70% of the fat present in freshly emerged flies was utilised during the next 4–5 days of maturation of adult flies (**Fig 2C**). Thus, excess larval fat could be retained in 5 day old DRL flies; another possibility is that less adult fat could be utilized during early adult stages, which could be responsible for higher triglyceride levels in mature flies. To check for retention of larval fat in mature DRL flies, we measured the mRNA levels of *fbp1* (*fat body protein1*), an ecdysone target gene expressed specifically in the larval fat body, as a marker for the presence of the larval fat body in adult flies [70]. The five day old DRL flies showed higher levels of *fbp1* transcripts providing proof that the excess stored fat, which is seen in adult flies, could be due to retention of the larval fat body **(Fig 3A)**. However, we did not observe the presence of larval fat cells in 5 day old DRL flies [71]. No such differences were seen in the *fbp1* transcript levels from pupal samples **(Fig 3B)**, which suggests that the difference in triglyceride levels in the pupal stage is mainly due to adult fat body, and excess larval fat body do not contribute to the difference in stored fat in pupal stages. Thus, the fat body from the larval stages was retained in the DRL flies due to the low utilization of larval fat during the early adult stages. The larval fat body could contribute to the obese phenotype observed in 5 day old DRL flies, in addition to the increase in lipid droplet size of the adult fat body **(Fig 1A)**. However, it is still possible that the excess fat in 5 day old DRL adults could also be due to enhanced triglyceride synthesis during early adult stages in addition to lower utilization of stored fat. Next, we checked whether the excess fat in DRL flies is due to the *de novo* synthesis and storage of fat in the adult stages.

Freshly emerged DRL flies have more stored fat when compared to control flies **(S1B Fig)**, hence we decided to equalize the fat content in both sets of flies by starving them continuously for 48 hours. We then subjected the fat depleted flies to normal feeding for five days and measured fat content to check if DRL flies synthesise and store more fat than control flies. DRL and control flies subjected to 48 hours of starvation led to a depletion of stored fat levels such that the levels of stored fat were equal in DRL and control flies. The fat stores in both sets of flies subjected to 48 hours of starvation upon re-feeding on normal food also remained the same **(Fig 3C)**. Thus, *de novo* synthesis of fat may not have led to the difference in fat levels between 5 day old DRL and control flies. To confirm this, we measured the expression of genes responsible for the generation of fat stores; *ACC* (Acetyl-CoA carboxylase), *FAS* (*fatty-acid synthase*) and *GPAT* (*Glycerol-3-phosphate acyltransferase*); to our surprise the levels of GPAT mRNA was found to be low, the levels of other triglyceride biosynthesis factors were found to be slightly low, but these changes were not significant, in the DRL animals than control **(Fig 3A)**. It may also be noted that five days old DRL fed less than control flies when subjected to starvation, implying that the obese phenotype observed is independent of feeding **(Fig 1D)**. Thus, the excess fat in DRL flies is not due to *de novo* synthesis of fat in the adult stages or not due to excess feeding in adult DRL flies. Obesity in mature DRL flies is due to higher fat levels inherited from pupal stages and due to low utilization of larval and adult fat during early adult stages. Next, we tried to identify the molecular mechanism that led to the adult metabolic effects in DRL flies.

### Reduced insulin signalling led to metabolic phenotypes in mature adult flies

**Fig 4:**
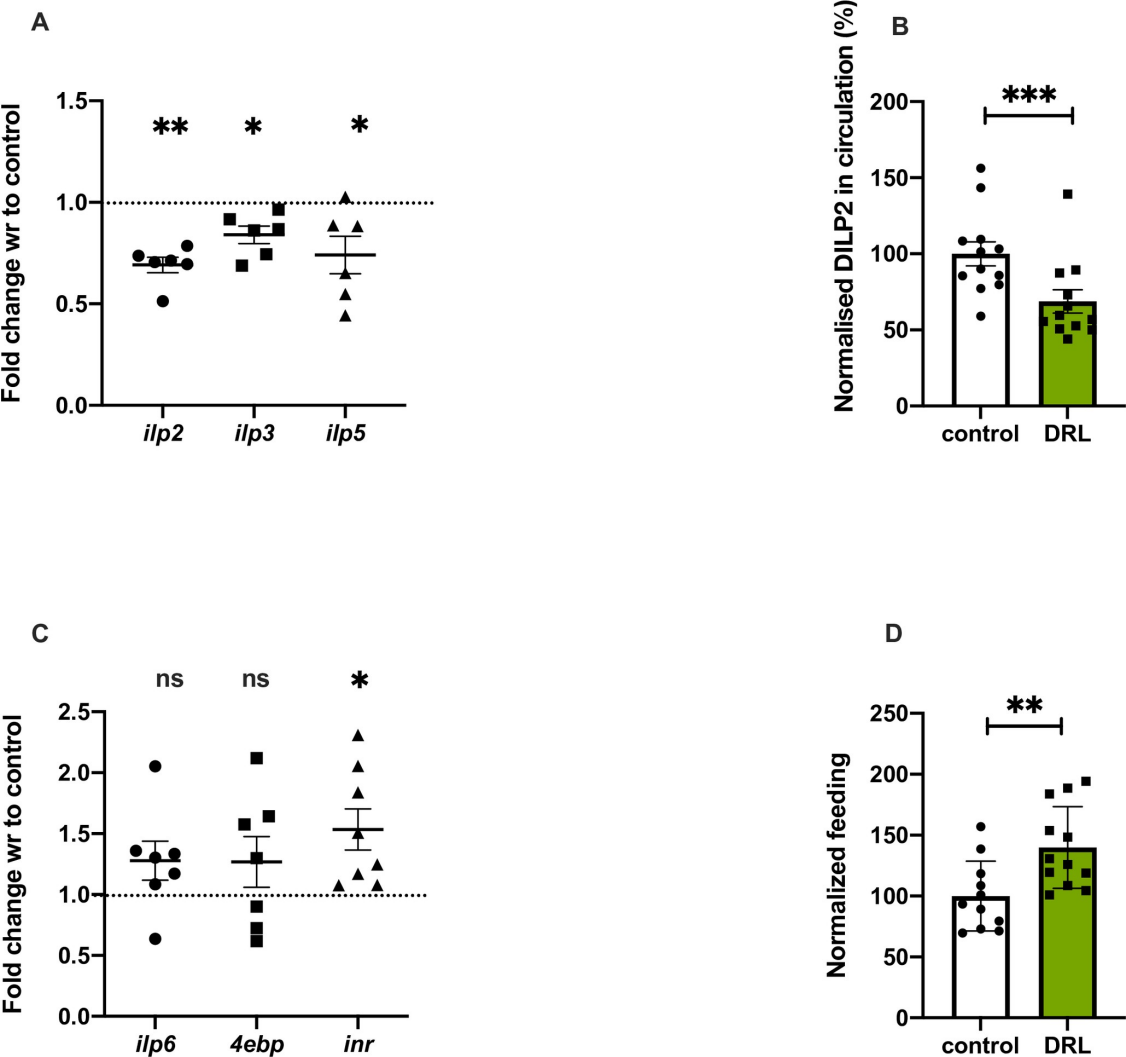
Nutrient restriction in larvae reduced insulin signalling. Nutrient restriction in larvae led to reduced insulin signalling. Shown is mRNA levels in 5-day old control and DRL flies, data are shown as fold change in mRNA levels, values are normalised to control flies and fold change in DRL flies is shown. (A) *dilp2* [p-value = 0.0005], *dilp3* [p-value = 0.0136] and *dilp5* [p-value = 0.0375] mRNA levels from 3rd instar larvae. [independent biological replicates = 6]. (B) DILP2 levels in the hemolymph of 3rd instar DRL and control larvae. [independent biological replicates = 12; p-value = 0.0095]. (C) *dilp6* [p-value = 0.1326], *4ebp* [p-value = 0.2445] and *inr* [p-value = 0.0158] mRNA levels from 3rd instar larvae. [independent biological replicates = 6;]. All transcripts levels were normalised with *rp49* mRNA (Unpaired Students t-test with Welch Correction). (D) Feeding is increased in DRL larvae compared to control larvae, data is shown as percentage feeding normalised to 100% control larvae [control n = 11; DRL n = 12, p-value <0.0058; Unpaired Students t-test with Welch Correction]. [p-value *<0.05; ** <0.*01*,*** <0.*001*, **** <0.*0001*, *data is presented as mean ± SEM*].

Insulin regulates various cell signalling and biochemical pathways which controls protein synthesis and maintain metabolic balance depending on the nutrient status of the organism. In *Drosophila*, insulin-like peptides (DILP2, 3 and 5) produced by IPCs control growth, metabolism, stress sensitivity, life span and feeding [58, 72, 73]. Based on the nutrient levels, *dilp* gene expression varies and DILP secretion from IPCs is regulated, in addition to this insulin signalling is controlled by other signalling mechanisms [41, 74, 75]. To measure the effects of larval nutrition on insulin signalling we measured the transcript levels of *dilp2*, *dilp3* and *dilp5*, which showed a reduction in response to low food levels **(Fig 4A)**. There was a significant reduction in the circulating levels of DILP2 in the hemolymph **(Fig 4B)**; however, DILP2 levels in the IPCs was not affected **(S2A and S2B Fig)**. In addition, systemic insulin signalling was measured by quantifying the expression of genes, which are under direct negative regulation by the insulin signalling pathway [76–78]. The mRNA levels of *inr*, *4ebp* and *dilp6* were found to be high in low fed larvae indicating a reduction in systemic insulin signalling **(Fig 4C)**. In addition, we observed enhanced feeding responses in the larvae **(Fig 4D)**, similar to responses reported earlier in food deprived larvae and also in response to low insulin signalling, due to its anorexigenic effects. Thus, gene expression of mNSC *dilps*, hemolymph levels of DILP2 and insulin signalling are low in larval stages in response to low nutrition diet, which is not unexpected as previous reports show a direct connection between nutrient environment and insulin signalling.

**Fig 5:**
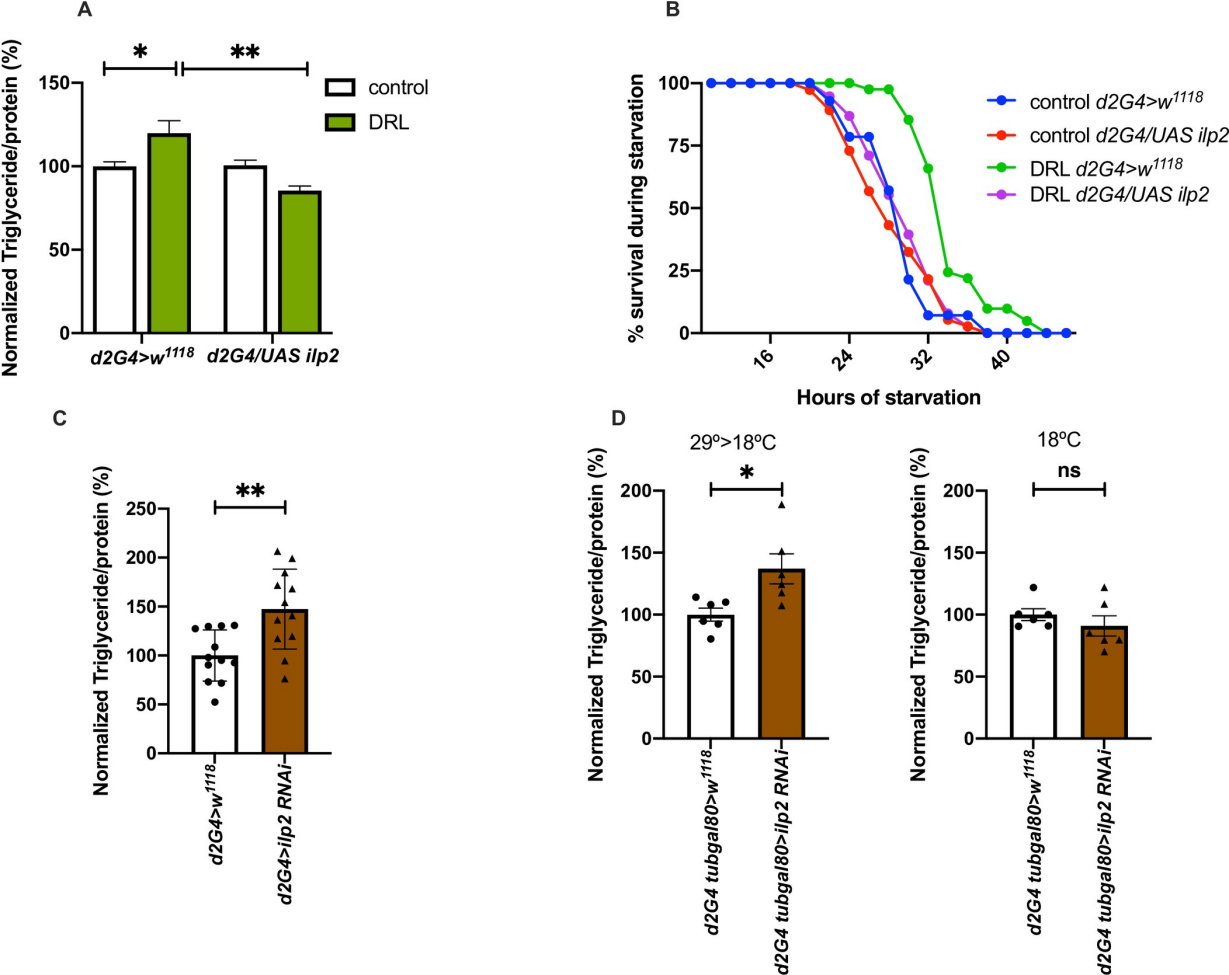
Reduced insulin signalling caused by nutrient restriction in larvae led to adult phenotypes. Reduced insulin signalling in larvae fed on low nutrients is responsible for the metabolic phenotypes. (A) Enhanced triglyceride levels in mature DRL flies is rescued by overexpression of *dilp2* in the IPCs. Data is represented as the ratio of triglyceride to protein levels in %; values are normalised to *dilp2Gal4>w*^*1118*^ fed normally during larval stages, the ratio of triglyceride to protein levels in % for *dilp2Gal4>w*^*1118*^ fed with 50% larval food during larval stages, *dilp2Gal4>UAS-dilp2* normally fed during larval stages and *dilp2Gal4>UAS-dilp2* fed with 50% larval food during larval stages are shown [independent biological replicates = 6; p-value between DRL *dilp2Gal4>w*^*1118*^ and DRL *dilp2Gal4>UAS-dilp2* is = 0.0043; Unpaired Students t-test with Welch Correction]. (B) Enhanced resistance to starvation in mature DRL flies is reduced by overexpression of *dilp2* in the IPCs. Data are shown as the percentage of flies which were alive at various time points of starvation, values are shown for *dilp2Gal4>w*^*1118*^ fed normally during larval stages, *dilp2Gal4>w*^*1118*^ fed with 50% larval food during larval stages, *dilp2Gal4>UAS-dilp2* normally fed during larval stages and *dilp2Gal4>UAS-dilp2* fed with 50% larval food during larval stages [independent biological replicates = 3; p-value between DRL *dilp2Gal4>w*^*1118*^ and DRL *dilp2Gal4>UAS-dilp2* is = 0.0001; Log-Rank test, *data is presented as mean*]. (C) Downregulation of *dilp2* was sufficient to phenocopy the effect of larval nutrition on adult triglyceride levels. Data is represented as the ratio of triglyceride to protein levels in %; values are normalised to *dilp2Gal4>w*^*1118*^ and fold change in *dilp2Gal4>UAS-dilp2-RNAi* normally fed during larval stages [independent biological replicates = 12; p-value between *dilp2Gal4>w*^*1118*^ and DRL *dilp2Gal4>UAS-dilp2-RNAi* is = 0.0031; Unpaired Students t-test with Welch Correction]. (D) Down regulation of *dilp2* in the larval IPCs was sufficient to phenocopy the effect of larval nutrition on adult triglyceride levels. *dilp2Gal4; tubGal80*^*ts*^*> w*^*1118*^ and *dilp2Gal4; tubGal80*^*ts*^*> UAS-dilp2-RNAi* normally fed during larval stages were maintained at 29°C till late larval stages and transferred to 18°C till adult stages. Data is represented as the ratio of triglyceride to protein levels in %; values are normalised to *dilp2Gal4; tubGal80*^*ts*^*> w*^*1118*^ and fold change in *dilp2Gal4; tubGal80*^*ts*^*> UAS-dilp2-RNAi* normally fed during larval stages [independent biological replicates = 6; p-value between *dilp2Gal4; tubGal80*^*ts*^ *>w*^*1118*^ and DRL *dilp2Gal4; tubGal80*^*ts*^ *>UAS-dilp2-RNAi* is = 0.0263; Unpaired Students t-test with Welch Correction]. Control experiments for the *Gal80ts* activity were maintained at 18°C from embryo till adult stages. [independent biological replicates = 6; p-value between *dilp2Gal4; tubGal80*^*ts*^ *>w*^*1118*^ and DRL *dilp2Gal4; tubGal80*^*ts*^ *>UAS-dilp2-RNAi* is = 0.3611]. [p-value *<0.05; ** <0.*01*,*** <0.*001*, **** <0.*0001*, *data is presented as mean ± SEM*].

Next, we tested if the reduction in insulin signalling during larval stages is responsible for the metabolic phenotypes observed in DRL adult flies. Previous reports show over-expression of *dilp2* alone is sufficient to rescue the phenotypes caused by reduced insulin signalling [73, 79]. Likewise, *dilp2* overexpression in the IPCs was sufficient to rescue the fat phenotype and enhanced survival to acute starvation in DRL mature adults **(Fig 5A and 5B)**. While *dilp2* expression specifically in the larval feeding stages using *tub-Gal80*^*ts*^ rescued the fat phenotype, enhancing *dilp2* levels in the adult stage alone did not affect the fat levels in DRL adult flies. Thus, reduced insulin signalling in the larvae fed on a low nutritive diet resulted in obesity and starvation resistance in adult stages. Furthermore, down-regulation of *dilp2* transcripts using RNAi in the IPCs was sufficient to phenocopy the effects on stored fat levels seen in DRL flies **(Fig 5C)**. Moreover, *dilp2* - RNAi expression specifically during larval stages in the IPCs was sufficient to phenocopy the effects on stored fat levels seen in DRL flies **(Fig 5D)**. However, *dilp2* down-regulation in DRL flies did not enhance the metabolic phenotypes any further than the effect caused by low calorie diet **(S3A Fig)**.

Thus, a low nutrient diet in larvae reduced insulin signalling, which led to an increase in adult fat levels and enhanced resistance to starvation. Next, we looked at gene expression changes in the adult flies impacted by larval nutrition, which controls adult metabolic status.

### Larval nutrient restriction affects the transcription of adult fat metabolism genes

**Fig 6:**
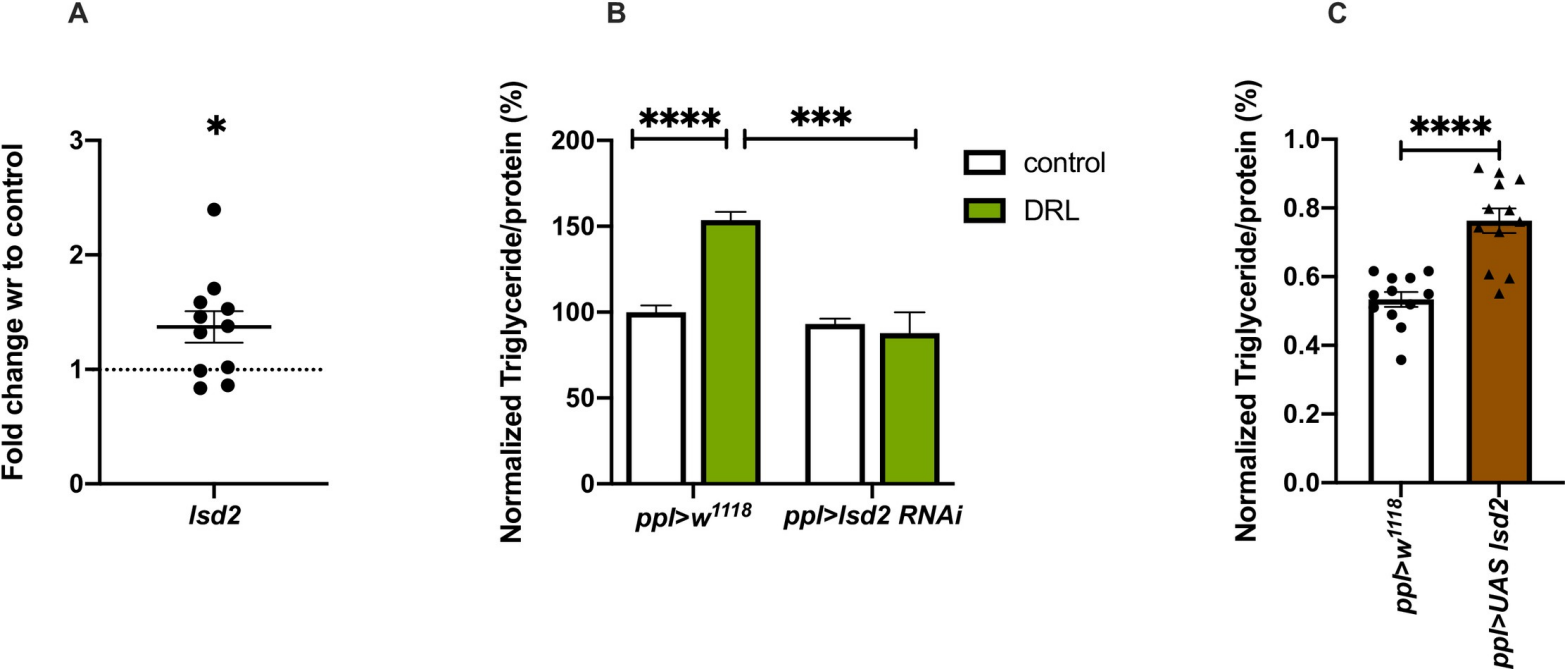
Nutrient restriction during larval stages affected the transcription of perilipin *lsd2*, which contributed to the adult phenotypes. Nutrient restriction in larvae led to reduced *lsd2* mRNA levels in the adult, which is responsible for the mature adult metabolic phenotypes. (A) Shown in the graph is *lsd2* mRNA levels in 5-day old control and DRL flies; values are normalised to control flies and fold change in mRNA levels in DRL flies is shown. [independent biological replicates = 11; p-value = 0.022; Unpaired Students t-test with Welch Correction]. (B) Enhanced triglyceride levels in mature DRL flies is rescued by *lsd2* RNAi. Data is represented as the ratio of triglyceride to protein levels shown in %; values are normalised to *pplGal4>w*^*1118*^ fed normally during larval stages; in comparison % ratio of triglyceride to protein levels are shown in *pplGal4>w*^*1118*^ fed with 50% larval food during larval stages, *pplGal4>UAS-lsd2-RNAi* normally fed during larval stages and *pplGal4>UAS-lsd2-RNAi* fed with 50% larval food during larval stages [independent biological replicates = 12; p-values between control fed *pplGal4>w*^*1118*^ and DRL *pplGal4>w*^*1118*^ is <0.0001; between DRL *pplGal4>w*^*1118*^ and DRL *pplGal4>lsd2 RNAi* = 0.0002; Unpaired Students t-test with Welch Correction]. (C) Overexpression of *lsd2* in normally fed larvae was sufficient to phenocopy DRL adult flies. Data is represented as the ratio of triglyceride to protein levels shown in %; values are normalised to *pplGal4>w*^*1118*^ and fold change in *pplGal4>UAS-lsd2* both genotypes were fed normally during larval stages [independent biological replicates = 12; p-values between *pplGal4>w*^*1118*^ and *pplGal4>UAS-lsd2* = 0.0001; Unpaired Students t-test with Welch Correction]. [p-value *<0.05; ** <0.*01*,*** <0.*001*, **** <0.*0001*, *data is presented as mean ± SEM*].

Fat storage is controlled by various enzymes that act in the metabolic pathway, a balance of their activity would determine storage and mobilisation of lipids in response to the energy needs of the organism. Larval nutrition could have affected genes that encode lipogenic and lipolytic enzymes, which might be responsible for the excess fat stores in DRL adults. We measured the expression of various lipogenic and lipolytic enzyme encoding genes in DRL flies. The levels of lipogenic genes are low in DRL flies **(Fig 3A)**, as discussed in the previous section; hence excess lipogenesis may not be responsible for the enhanced fat stores in adult flies. However, we found changes in the expression of genes that regulate lipolysis, which could be responsible for the DRL metabolic phenotypes. The levels of *lipid storage droplet 2* (*lsd2*) mRNA, a key player in regulating fat storage in *Drosophila*, was found to be enhanced in mature DRL flies **(Fig 6A)**. Lsd2, a *Drosophila* perilipin-like molecule, acts as a barrier between lipid droplets and lipases, restricting the activity of lipases on lipid droplets, and prevent mobilization of fat [56, 80, 81]. Enhanced levels of Lsd2 in DRL flies could be responsible for the excess fat storage, to check this we reduced *lsd2* expression levels by RNAi. Downregulation of *lsd2* transcript levels in the fat body rescued the excess fat storage phenotype in DRL flies **(Fig 6B)**. In addition, overexpression of *lsd2* in the fat body was sufficient to phenocopy DRL flies in the absence of any dietary manipulations in the larvae **(Fig 6C)**. However, the levels of *lsd2* was not affected in larvae in response to low larval feeding and manipulation of *lsd2* levels specific to larval stages did not affect adult fat stores or starvation responses **(S3B Fig)**. These results demonstrate that enhanced *lsd2* gene expression in mature adult flies resulting from reduced larval nutrition led to obesity, and increasing the levels of *lsd2* was sufficient to cause excess fat storage and starvation resistance in adult flies.

**Fig 7:**
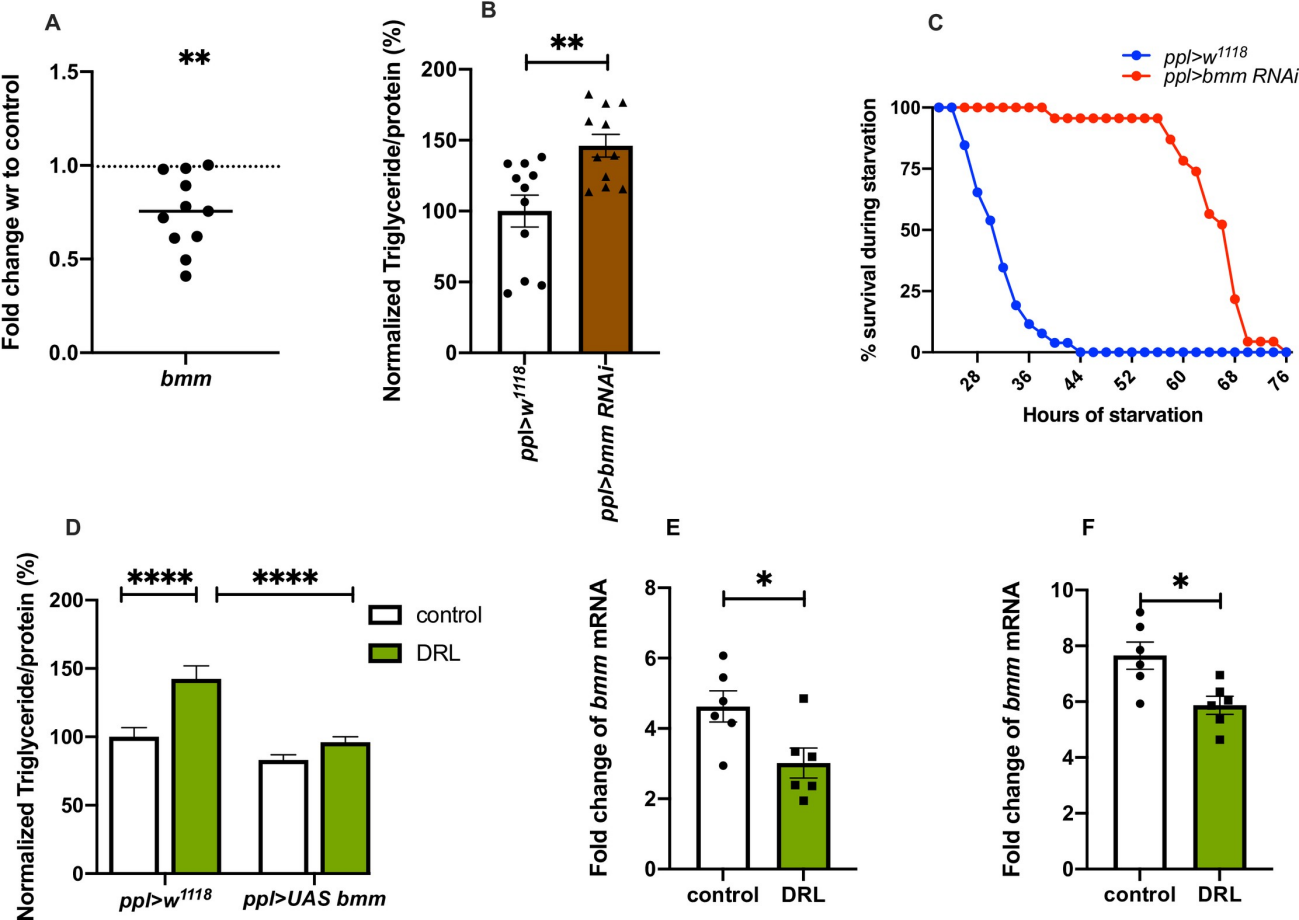
Nutrient restriction during larval stages led to reduced expression of lipase *brummer* which led to the phenotypes seen in adult flies. Nutrient restriction in larvae led to reduced *bmm* mRNA levels in the adult, which is responsible for the adult metabolic phenotypes. (A) *bmm* mRNA levels in 5-day old control and DRL flies are shown, values are normalised to control flies, and fold change in mRNA levels in DRL flies is shown. [independent biological replicates = 11; p-value = 0.0022; Unpaired Students t-test with Welch Correction]. (B) Downregulation of *bmm* in adult flies that emerged from normally fed larvae was sufficient to phenocopy DRL adult stored fat levels. Data is represented as the ratio of triglyceride to protein levels shown in %; values are normalised to *pplGal4>w1118* and fold change in *pplGal4> UAS-bmm-RNAi*, both conditions were fed normally during larval stages [independent biological replicates = 11; p-values between *pplGal4>w1118* and *pplGal4> UAS-bmm-RNAi* = 0.0083; Mann-Whitney test]. (C) Downregulation of *bmm* in adult flies that emerged from normally fed larvae was sufficient to phenocopy DRL adult starvation resistance. Data are shown as the percentage of flies which were alive at various time points of starvation; values are shown for *pplGal4>w1118* and fold change in *pplGal4>UAS-bmm-RNAi*, both conditions were fed normally during larval stages [independent biological replicates = 3; p-values between *pplGal4>w1118* and *pplGal4>UAS-bmm-RNAi* = 0.0001; Log-rank test, data is presented as mean]. (D) Enhanced triglyceride levels in mature DRL flies is rescued by bmm overexpression. Data is represented as the ratio of triglyceride to protein levels shown in %, values are normalised to *pplGal4>w1118* fed normally during larval stages fold change in *pplGal4>w1118* fed with 50% larval food during larval stages, *pplGal4>UAS-bmm* normally fed during larval stages and *pplGal4>UAS-bmm* fed with 50% larval food during larval stages [independent biological replicates = 30; p-value between Control *ppl>w1118* and DRL *ppl>w1118* is <0.0001 and p-value between DRL *ppl>w1118* and DRL *ppl>UAS-bmm* is = 0.0001; Mann-Whitney test]. Induction of *bmm* gene expression in response to acute starvation is attenuated in DRL flies. *bmm* mRNA levels in control and DRL flies subjected to 12 hours of starvation (E) and 24 hours of starvation (F) is shown, values are plotted as fold changes in mRNA levels in control and DRL flies in response to 12 and 24 hours of starvation. [independent biological replicates = 6; p-value for 12 hours starvation = 0.0257; p-value for 24 hours starvation = 0.0145; Unpaired Students t-test with Welch Correction]. [p-value *<0.05; ** <0.01,*** <0.001, **** <0.0001, data is presented as mean ± SEM].

In normal conditions, stored fat is mobilised by the activity of lipases for various biological needs. Brummer, a patatin-like domain containing adipocyte triglyceride lipase (ATGL), has been shown by earlier reports to play a key role in the breakdown of triglycerides in flies [54]. In response to starvation, the levels of *brummer* have been shown to increase, which aids in mobilising stored fat during nutrient deprivation [54, 55, 82]. We hypothesised that the levels of *brummer* gene expression is low in the DRL animals, which may lead to the storage of excess fat in the adults. The levels of *brummer* (*bmm*) mRNA was found to be low in DRL flies when compared to control in early and mature adult flies **(Fig 7A, S3C Fig)**. This observation suggests that the accumulation of stored fat due to reduced *bmm* gene expression could be responsible for the DRL adult fat phenotype. To test this, we downregulated *bmm* transcript levels in the fat body in flies that emerged from larvae fed normally, which phenocopied the effects seen on fat levels and starvation in DRL flies **(Fig 7B and 7C)**. However, reducing *bmm* levels in DRL flies showed a further increase in starvation resistance than DRL treatment alone **(S3E Fig)**. Thus, reduction of *bmm* gene expression induced by low larval diet and RNAi treatment led to an additive effect on starvation resistance. We also observed a rescue of the fat phenotype and starvation responses of DRL flies by restoring *bmm* levels in the fat body **(Fig 7D, S3D Fig)**. Further, starvation-induced expression of *bmm* was attenuated in DRL flies, which could be responsible for slow mobilization of stored fat and enhanced survival in response to acute starvation (**Fig 7E and 7F**). Together, these experiments suggest that reduced *bmm* levels and lower induction of *bmm* gene expression in response to starvation in the DRL flies could be responsible for the adult metabolic phenotypes. Even though, *bmm* gene expression in larvae did not show any changes in response to the low nutrient larval diet, reducing *bmm* levels specific to larval fat body led to a phenocopy of DRL adults **(S3B Fig)**. Thus, mature flies that emerge from larvae that were reared on a restricted diet were obese as they utilised less fat during early adult stages. The DRL flies mobilised less amount of stored fat, which improved their survival, during extended starvation. We summarize that these metabolic effects in DRL flies are due to lower levels of the ATGL Brummer and concomitant higher levels of the perilipin-like protein Lsd2.

## Discussion

Homeostasis is a remarkable capability of all living organisms, which aids them to withstand large changes in environmental conditions and function normally. For the maintenance of homeostasis, quick sensing of changes in the environmental conditions and efficient responses activating various signalling mechanisms are needed. Further, the nutritional status determines the energy expenditure for biological functions. Complex macromolecules obtained during feeding enter metabolic pathways, which leads to energy generation and synthesis of biomolecules; eventually, excess nutrients are stored in specialised tissues. Excess nutrients stored during plentiful food supply is used during starvation or during functions that require excess energy—like immune responses against infections and long-distance migration. A breakdown of the nutrient balance leads to severe metabolic disorders like obesity and diabetes.

A constant supply of nutrients throughout the life cycle of an organism plays a crucial role in performing normal biological functions. Variations in food availability are tolerated to a large extent, with the aid of homeostatic responses. However, the quality of diet during certain stages of life can have major influences on other stages [17, 19]. The long-term physiological consequences of early developmental experiences have been properly documented only recently. Maternal and developmental diet quality has a deep impact on the development of the embryo and also during the mature life stages [20, 67, 68]. The influence of early nutritional environment on human health is clearly seen in the case of ‘Dutch hunger winter,’ where individuals who have been *in utero* during a period of scarce food availability showed higher mortality rates, metabolic disorders, and behavioural problems during advanced stages of their life [65, 66]. In passerine birds, evidence from both lab and wild suggest that early developmental conditions influence fecundity [67]. The nestling body conditions and other environmental factors of migratory tits determined the amount of subcutaneous fat, which impacted the success of their migration [68]. In *Drosophila*, acute starvation of third instar larvae led to the production of adults with fewer ovarioles [20]. Other studies in flies showed that larval dietary yeast influenced adult body size, fecundity and ovariole number [20, 64]. The larval diet‐restriction phenotypes were similar to the effects of insulin pathway mutations; hence regulation of insulin signalling by larval diet could be mainly responsible for the effects seen in adult flies [44, 45, 47]. The effects of low nutrition during early life stages may constrain growth and developmental processes, giving rise to offsprings, which are less fit.

The external conditions during development can also shape the individual in such a way as to better prepare it for the environmental conditions it is most likely to encounter during its adult life. Recent reports suggest that developmental nutrition may influence the metabolic status of adult stages, thus preparing the adult organism to anticipate a similar nutritional environment. The influence of larval nutrition on adult nutrient stores and the adult flies’ responses to acute depletion of food reserves has not been studied thus far, though larval diet influenced ageing and fecundity in adult flies [18, 20, 63]. Defects in insulin signalling led to reduced adult body size and excess fat storage [44, 47, 62, 83]. However, in these cases insulin signalling is affected from the embryo to adult stages. It is not clearly understood whether the effects on adult fat levels are due to reduced insulin signalling in the developmental stages or the adult mature stages. Certainly, these papers do not talk about larval nutrition affecting adult lipid levels. We were interested in studying the impact of nutrition during larval growth stages on the metabolic status of adult flies. Here, we show that dilution of normal larval food by half led to metabolic phenotypes in adults. The food was diluted by 50% as this condition did not create any strong developmental delays or had minimal effects on the body size of the adult flies eclosed from these larvae. Also, we subjected the entire larval stages to low diet treatment to cover the growth period. To our surprise, we saw that nutrient resources, especially the triglyceride levels, in the mature adult flies, were higher in response to low larval feeding **(Fig 1A)**. The size of lipid droplets were increased in the abdominal fat body of these flies. As a result of increased fat stores, we also report enhanced resistance to acute starvation-induced stress and hunger induced feeding responses in adult flies **(Fig 1B and 1D)**. Thus, exposure to reduced nutrition during larval stages, led to enhanced nutrient stores, which influenced the adult flies’ responses to acute depletion of food reserves and aid in their better survival. By choosing larval food conditions that do not affect growth, the adult metabolic effects we observe is independent of developmental growth regulation. Low diet levels affected insulin signalling in the larvae and increasing insulin signalling in the larvae fed with diluted food rescued the metabolic phenotypes seen in the adults **(Figs 4 and 5)**. Reduced insulin signalling in normally fed larvae was sufficient to show phenotypes in adult flies that emerged from normally fed larvae, which were similar to DRL flies **(Fig 5C)**. However, the reduction of *dilp2* mRNA levels below what is achieved by a low calorie diet does not show an additive effect on starvation resistance **(S3A Fig)**. Thus, diet influenced insulin signalling in the larvae and determined the amount of stored fat levels and starvation responses in the adults, which is independent of the effect of larval nutrition on growth and development. The effect of larval nutrition on adult nutrient stores may also be responsible for the effects seen on reproductivity and ageing; however, this has not been tested yet. Thus, developmental nutrition influenced the metabolic status of the adult flies, preparing the adult organism to anticipate a similar nutritional environment.

Further, our analysis to identify the source of excess stored fat in response to low larval nutrition showed the presence of the larval fat body in mature adult flies, which is normally utilized beforehand. Energy stores established during larval stages are allocated for fuelling wandering larvae, pupal development and early adult stages. Larval tissues undergo autophagic cell death in pupal stages, whereas, the larval fat body, which is refractive to cell death is preserved in pupal and early adult stages as dispersed fat cells [69]. The larval fat cells are consumed in the early adult flies, which provides support for somatic maintenance and reproduction [71]. Nutrients acquired from the larval fat cells have been shown to contribute to adult stress resistance and drive ovarian development [64, 71]. Here, by using a marker for the larval fat body - *fbp1* mRNA, we show that larval fat body is retained in 5 days old DRL adult flies and contribute to excess fat storage **(Fig 3A)**. We did not find any evidence for enhanced lipogenesis in the DRL flies **(Fig 3A)**. Previous reports have shown that newly eclosed flies contain larval fat cells and are resistant to starvation in comparison to older flies, blocking cell death of the larval fat cells also made flies resistant to starvation. Similarly, the excess larval fat body present, in addition to the excess fat stores seen in the adult fat body, in 5 day old DRL adults may have rendered the flies resistant to acute starvation. We are yet to determine whether diet restriction during larval stages made the larval fat cells refractive to cell death in early adult flies.

What could be the reasons behind the metabolic phenotypes seen in adult flies that emerge from low fed larvae? Mature DRL flies are obese and are impaired in their ability to mobilize lipids in response to acute food deprivation. However, these flies show resilience against starvation as they utilize stored fat at a much slower rate, but the total amount of lipids mobilized in response to 24 hours of starvation is more than the control flies, due to excess stored fat present. We performed a candidate gene expression study to look at lipid metabolic pathway genes that could be affected in DRL flies. Interestingly, the mRNA levels of *Drosophila* ATGL *brummer* were low and *Drosophila* perilipin *lsd2* was high in DRL flies **(Figs 6A and 7A, S3C Fig)**. *Drosophila* lipolytic machinery consists of Brummer lipase, responsible for most of the basal and fasting-induced lipolysis, regulated likely via transcriptional control [54, 55, 82]. Like the DRL flies, *brummer* mutant flies are obese, show larger lipid droplets in the adult fat body and show impaired lipid mobilization in response to food deprivation [54]. Freshly eclosed *bmm* mutant adults are mildly obese, whereas mature *bmm* mutant flies have more stored fat, which is comparable to the phenotype of DRL flies. The *bmm* mutants show extended survival under food deprivation by slower metabolization of stored fat than controls, also similar to DRL flies. Induction of *bmm* expression levels in response to fasting was low in DRL flies, which could explain the slow mobilization of triglycerides **(Fig 7E)**. Thus, the phenotypes seen in DRL flies are reminiscent of *bmm* mutants. Finally, using genetic means, we show that defects in fat metabolism and starvation responses of DRL flies are due to low *bmm* levels **(Fig 7)**.

*Drosophila* Lsd2 acts as a negative regulator of lipid hydrolysis, similar to perilipin function in mammals. The *lsd2* mutant flies contain significantly low triglyceride levels and overexpression of Lsd2 led to obesity; the obese Lsd2 overexpressing flies survived better during starvation [56]. Mature DRL flies are similar in starvation resistance and obese phenotypes to Lsd2 overexpressing flies. The reduction of high *lsd2* levels using RNAi rescued the metabolic phenotypes in DRL flies **(Fig 6)**. We also observe that despite the changes in insulin signalling in response to reduced nutrient levels in the larvae, the expression of *bmm* and *lsd2* did not show any changes. Thus, the effects of low larval food is reflected on fat utilization genes of the adults and not larvae.

Collectively, these results indicate that larval nutrition affects adult Brummer and Lsd2 levels and thereby organismal fat stores and starvation stress responses. Although the exact molecular mechanism which led to changes in gene expression of these fat storage regulators is yet to be discerned, earlier studies have pointed towards the effect of larval nutrition on genome-wide transcriptional changes. Thus, low nutritional availability in the developing organism leads to adaptive changes that prepare the adults in anticipation of a scarce nutrient environment.

## Materials and methods

### Reagents and fly strains

Reagents and fly strains used were as follows: *w*^*1118*^ (#3605), *ppl-Gal4* (#58768) and *tubGal80*^*ts*^ (#7017) were obtained from Bloomington Drosophila Stock Center (https://bdsc.indiana.edu), *dilp2-Gal4* and *UAS-dilp2* [41]. *dilp2Gal4; tubGal80*^*ts*^ [79] and *pplGal4; tubGal80*^*ts*^ lines were generated in the lab. *UAS-bmm* and *UAS-lsd2* were provided by Dr. Ronald Kühnlein. All RNAi lines were procured from Vienna Drosophila RNAi Center (VDRC).

### Experimental setup

Drosophila control media contains cornmeal (5.82%), dextrose (5.08%), inactive yeast (2.36%), (agar 0.8%) and nipagin (10% w/v in ethanol) in the normal fed condition. 50% Larval media (DRL) contains cornmeal (2.91%), dextrose (2.54%) inactive yeast (1.18%), while agar and nipagin were the same as control. All other diluted food conditions were made from the normal fed food conditions above mentioned keeping the agar level constant. All flies were reared at 25°C (unless specified otherwise) under 70% humidity with 12:12 light-dark cycle in a Percival Drosophila growth chamber DR 36VL. Age-matched flies were used for all metabolic assays. Drosophila embryo cages were set up and first instar larvae were collected within 2–3 hours of egg hatching. Fifty larvae were collected into fresh vials with specific media and larvae of specific genotype were selected using GFP balancers with the help of a Zeiss Stereo Discovery V20 Fluorescence microscope. Multiple independent collections were made. Late non-wandering third instar larvae were collected for qRT-PCRs. Fresh pupae collected within an hour of pupation and aged for 24 hours were collected for fat measurements and other assays. Freshly eclosed flies within 2 hours of eclosion or aged for five days in standard media were collected for metabolic measurements and starvation sensitivity assays. Adult males were collected immediately upon eclosion and batches of 10 flies were transferred into fresh normal food vials; assays were performed on corresponding days. For larval specific expression of transgenes the crosses were maintained at higher temperature of 29°C (restrictive temperature for temperature sensitive Gal80) to allow Gal4 expression, during larval stages, and switched to lower temperature of 18°C to repress Gal4 expression after larval stages (permissive temperature for temperature sensitive Gal80). Control experiments for temperature sensitive Gal80 were performed by maintaining crosses at a lower temperature of 18°C to block Gal4 expression throughout developmental and adult stages,

### Triglyceride and glycogen measurements

Batches of 5 adult male flies of ages mentioned in the main text were collected and homogenised using Bullet Blender Storm using Zirconium Oxide 1.0 mm beads (Next Advance, NY). Heat inactivation of the samples was carried out at 70°C for 5 minutes, following which the samples were centrifuged at 14000 rpm for 4 min. Triglyceride and protein levels were measured using the Sigma Triglyceride kit and Bio-Rad protein assay reagent, respectively, following the manufacturers’ instructions. Optical Density (OD) was measured by colorimetric analysis using TECAN Infinite M200 pro multi-mode plate reader in 96 well plates. Sample preparation for glycogen measurement was the same as given above with the further assay being carried out using Sigma glycogen assay kit, following the manufacturers’ protocol. Independent biological replicates of this experiment were performed; the number of replicates is available in the figure legends.

### Weight measurement

Sets of ~30 five day old flies were kept in a 0.6 μl tube and weighed using Sartorius Cubis® Micro Balance. The average weight of each fly was calculated. Independent biological replicates of this experiment were performed, the number of replicates is available in the figure legends.

### Feeding assays for larvae and adult flies

First instar larvae were transferred to both Control and DRL Orange G coloured food vials. Early third instar larvae were harvested for assays.

Both five day old DRL and control flies, 15 flies per set, were transferred to 1% agar vials for 12 hours and the control flies were provided with yeast paste. Following this 12 hour period, the feeding assay was carried out by transferring them to vials containing yeast paste with Orange G (Sigma) for 30 minutes.

The flies and larvae were then homogenized using zirconium oxide beads in homogenization buffer (0.05% Tween 20) using the bullet blender, and the supernatant was used for the colorimetric estimation at 478 nm using TECAN Infinite M200 pro multi-mode plate reader in 96 well plates. The absorbance was plotted normalized against the control. Detailed protocol can be found in [79]. Independent biological replicates of this experiment were performed, the number of replicates is available in the figure legends.

### Glucose measurement

Glucose measurement was carried out using Glucose Assay kit (Sigma GA2020). Hemolymph from 30 flies aged 5 days in normal fly food was collected using Zymo Spin columns by pricking the thorax with a fine needle in ice-cold conditions by centrifugation at 15,000 rpm for 15 minutes. 1μl of hemolymph was added to the Glucose Assay Reagent and incubated at 37°C for 30 minutes. The reaction was stopped by adding 6M H2SO4. Absorbance was measured at 540 nm using a F200 TECAN 96 well micro-plate reader. Independent biological replicates of this experiment were performed; the number of replicates is available in the figure legends.

### Starvation sensitivity

Flies (~15 flies per vial) were transferred to 1% agar vials, and the number of dead flies was marked every two hours. The percentage of flies died at various time points was calculated and subtracted from total flies and plotted as % survival. Independent biological replicates of this experiment were performed; the number of replicates is available in the figure legends.

### Dilp2 antibody staining

Dilp2 polyclonal antibody was raised using a peptide corresponding to amino acids 108–118 of the Dilp2 sequence (TRQRQGIVERC) as an immunogen in rabbits (Eurogentec, Belgium). Third instar crawling larval brains were dissected in ice-cold PBS and fixed in 4% paraformaldehyde (PFA) (Sigma) for 20 min at RT. Primary antibody anti-DILP2 at a dilution of 1:200 was added and incubated overnight at 40C in blocking buffer, BBT (1% BSA with PBT). Samples were then incubated with secondary antibody Alexa Fluor® 568 Goat Anti-Rabbit IgG (dilution of 1:1000) in BBT for not more than 2 h at room temperature. Samples were then mounted in mounting medium (SlowFade® Gold Antifade Reagent with DAPI, Invitrogen).

### Enzyme-linked immunosorbent assay (ELISA)

For hemolymph extraction, 10 third instar larvae were punctured using a fine needle and centrifuged using Zymo-Spin columns at 4°C—15,000 rpm to collect hemolymph extracts [84]. Hemolymph extracts were diluted with phosphate-buffered saline PBS (1:50). About 50 μl of each of the samples was coated into the 96 well EIA plate (Costar) and incubated at room temperature overnight. The plates were then blocked using the blocking buffer, BBT (1% BSA with PBT). Following this, the plates were washed twice with PBT (PBS.1%Tween20) and primary antibody DILP2 (1:2000) was added to each of the well plates and incubated overnight at 40C. The plates were washed using PBT, following which the secondary antibody, which is anti-Rb IgG conjugated with HRP (1:2500) and incubated for 2 h at room temperature. The plates were washed with PBT followed by the addition of TMB (Himedia) substrate and incubated in room temperature for 10–20 minutes and the reaction was stopped by adding 100 μl of 2N H2SO4 to each well and the absorbance was measured colorimetrically at 540 nm. Independent biological replicates of this experiment were performed; the number of replicates is available in the figure legends.

### Nile red staining

For lipid droplet staining in adult fat body, abdomens of 10 (5-day old) flies were dissected in ice-cold Shields and Sang medium and fixed in 4% formaldehyde in PBT for 30  minutes at room temperature. After fixation, the samples were washed twice in 1X PBT for 10 min each and incubated with freshly prepared 1:50000 dilution of Nile Red (Cat # 72485) in acetone for 5 min. Subsequently, tissue was rinsed twice with autoclaved Milli-Q and mounted.

### Quantitative PCR (qPCR)

Batches of 5 flies or 5 third instar larvae were flash-frozen in liquid nitrogen and total RNA was extracted using Qiagen RNeazy kit method (74134) with Genomic DNA eliminator spin column as per manufacturers instructions. Reverse transcription reaction was carried out using oligo-dT primers and SuperScript RT-III (Invitrogen) in the ABI proflex PCR system and the synthesised cDNAs were used for qPCR with BioRad CFX96. Independent biological replicates of this experiment were performed; the number of replicates is available in the figure legends.

The following primers were used:

dilp2 FP    5’-GGCCAGCTCCACAGTGAAGT-3’

dilp2 RP    5’-TCGCTGTCGGCACCGGGCAT-3’

dilp3 FP    5’-CCAGGCCACCATGAAGTTGT-3’

dilp3 RP    5’-TTGAAGTTCACGGGGTCCAA-3’

dilp5 FP    5’-TCCGCCCAGGCCGCAAACTC-3’

dilp5 RP    5’-TAATCGAATAGGCCCAAGGT-3’

dilp6 FP    5’-CGATGTATTTCCCAACAGTTTCG-3’

dilp6 RP    5’-AAATCGGTTACGTTCTGCAAGTC-3’

rp49 FP    5’-GCTAAGCTGTCGCACAAA-3’

rp49 FP    5’-TCCGGTGGGCAGCATGTG-3’

bmm FP    5’-ACGCACAGCAGCGACATGTAT-3’

bmm RP    5’-CTTTTCGCTTTGCTACGAGCCC-3’

lsd2 FP    5’-GAATGGCAAGAGTTCTGA-3’

lsd2 RP    5’-GACAGAAATACCGTCGAG-3’

4ebp FP    5’-CACTCCTGGAGGCACCA-3’

4ebp RP    5’-GAGTTCCCCTCAGCAAGCAA-3’

gpat FP    5’-CTGATCTCCACTAGCACTGCG-3’

gpat RP    5’-CTGATCTCCACTAGCACTGCG-3

fas FP    5’-GTTGGGAGCGTGGTCTGTAT-3’

fas RP    5’-GGTTTAGGCCAGCGTCAATA-3’

inr FP    5’-AACAGTGGCGGATTCGGTT-3’

inr RP    5’-TACTCGGAGCATTGGAGGCAT-3’

acc FP    5’-CCGGTAGCTCTGCATCATCT-3’

acc RP    5’-AGACCACCACCAAAGTGTCC- 3’

fbp1 FP    5’-GCTGGGAATTGATTCGGATTT-3’

fbp1 RP    5’-CAGCTGGTCGCACGTCTTAAC-3’

## Supporting information

S1 Fig (A) Triglyceride levels are higher in DRL pre-pupae in comparison with control pre-pupae, data is shown as percentage ratio of triglyceride to total protein levels, normalised to 100% control pre-pupae [independent biological replicates = 6; p-value = 0.0001; Unpaired Students t-test with Welch Correction]. (B) Triglyceride levels are higher in DRL freshly eclosed flies when compared to control flies, data is shown as percentage ratio of triglyceride to total protein levels, normalised to 100% control flies [independent biological replicates = 28; p-value = 0.0296; Unpaired Students t-test with Welch Correction]. (C) Glycogen content remains unchanged in 5 day old DRL and control flies, data is shown as percentage glycogen content normalised to 100% control flies [independent biological replicates = 12; p-value = 0.1007; Unpaired Students t-test with Welch Correction]. (D) Glucose content shows no significant difference in 5 day old DRL and control flies, data is shown as percentage glucose level normalised to 100% control flies [control n = 16; DRL n = 18; p-value = 0.7984; Mann-Whitney test]. (E) Triglyceride levels are higher in mature flies that emerged from larvae fed with 75%, DRL and 25% diluted food, data is shown as percentage ratio of triglyceride to total protein levels, normalised to 100% control flies [independent biological replicates n = 9; p-value between control and 75% is = 0.0097, Unpaired Students t-test with Welch Correction; between control and DRL is = 0.0004 Unpaired Students t-test with Welch Correction, and between control and 25% is = 0.0315; Mann-Whitney test]. [p-value *<0.05; ** <0.01,*** <0.001, **** <0.0001, data is presented as mean ± SEM].
(TIF)

S2 Fig (A) DILP2 staining of IPCs in *3^rd^* instar larvae of control and DRL. (B) Corrected Total Cell Fluorescence levels of control and DRL in IPCs, data is shown as percentage of CTCF content normalised to 100% in control larvae [independent biological replicates = 11; p-value = 0.6992; Unpaired Students t-test with Welch Correction]. [p-value *<0.05; ** <0.01,*** <0.001, **** <0.0001, data is presented as mean ± SEM].
(TIF)

S3 Fig (A) Enhanced resistance to starvation in mature DRL flies is not affected by down regulation of *dilp2* mRNA in the IPCs, data are shown as the percentage of DRL flies of the genotypes *dilp2Gal4>w^1118^* and *dilp2Gal4>UAS-dilp2-RNAi*, which were alive at various time points of starvation, values are shown for *dilp2Gal4>w^1118^* and *dilp2Gal4>UAS-dilp2-RNAi* flies fed on 50% diet during larval stages [independent biological replicates = 3, p-value between DRL *dilp2Gal4>w^1118^* and *dilp2Gal4>UAS-dilp2-RNAi* flies is = 0.0011; Log-rank test]. (B) Over expression of *lsd2* in the larval stages alone did not make any significant change in the fat levels in mature adults. *pplGal4*; *tubGal80^ts^> w^1118^* and *pplGal4; tubGal80^ts^>* UAS-*lsd2* normally fed during larval stages were maintained at 29°C till late larval stages and transferred to 18°C till adult stages. [independent biological replicates = 12; p-values between *pplGal4; tubGal80^ts^*> *w1118* and *pplGal4; tubGal80^ts^*> UAS-*lsd2* = 0.0649; Unpaired Students t-test with Welch Correction]. Control experiments for *tubGal80^ts^* were maintained at 18°C from embryo till adult stages. [independent biological replicates = 12; p-values between *pplGal4; tubGal80^ts^> w1118* and *pplGal4; tubGal80^ts^*> UAS-*lsd2* = 0.9313]. Down regulation of *bmm* in the larval stages alone can significantly increase the fat levels in mature adults. *pplGal4; tubGal80^ts^> w^1118^* and *pplGal4; tubGal80^ts^>* UAS-*bmm* RNAi normally fed during larval stages were maintained at 29°C till late larval stages and transferred to 18°C till adult stages. [independent biological replicates = 12; p-values between *pplGal4; tubgal80*>*w^1118^* and *pplGal4; tubgal80*> *UAS*-*bmm RNAi* = 0.0013; Unpaired Students t-test with Welch Correction]. Control experiments for *tub-Gal80^ts^* activity were maintained at 18°C from embryo till adult stages. [independent biological replicates = 12; p-values between *pplGal4; tubgal80*>*w^1118^* and *pplGal4; tubgal80*> *UAS*-*bmm RNAi* = 0.0640].(C) *bmm* levels in control and DRL freshly eclosed flies normalised to *rp49* mRNA level, values are normalised to control flies and fold change in mRNA levels in DRL flies is shown [independent biological replicates = 7; p-value = 0.0001; Unpaired Students t-test with Welch Correction]. (D) Enhanced resistance to starvation in mature DRL flies is reduced by overexpression of *bmm* in the fat body, data are shown as the percentage of flies of control and DRL, which were alive at various time points of starvation, values are shown for *ppl>w^1118^* fed normally during larval stages, *ppl>w^1118^* fed with 50% larval food during larval stages, *ppl>UAS-bmm* normally fed during larval stages and *ppl>UAS-bmm* fed with 50% larval food during larval stages [independent biological replicates = 3, p-value between DRL *bmm* over expression and control flies is = 0.0001; Log-rank test]. (E) Enhanced resistance to starvation in mature DRL flies is further enhanced by down regulation of *bmm* mRNA in the IPCs, data are shown as the percentage of DRL flies of the genotypes *pplGal4>w^1118^* and *pplGal4>UAS-bmm-RNAi*, which were alive at various time points of starvation, values are shown for *pplGal4>w^1118^* and *pplGal4>UAS-bmm-RNAi* flies fed on 50% diet during larval stages [independent biological replicates = 3, p-value between DRL *pplGal4>w^1118^* and *pplGal4>UAS-bmm-RNAi* flies is = 0.0001; Log-rank test]. [p-value *<0.05; ** <0.01,*** <0.001, **** <0.0001, data is presented as mean ± SEM].
(TIF)

S1 Data (XLSX)
