# Supplementary figures and images for: Larval nutrition influences adult fat stores and starvation resistance in Drosophila
Source: PLoS One. 2021 Feb 19;16(2):e0247175. doi: 10.1371/journal.pone.0247175 (PMC7895371; doi:10.1371/journal.pone.0247175)

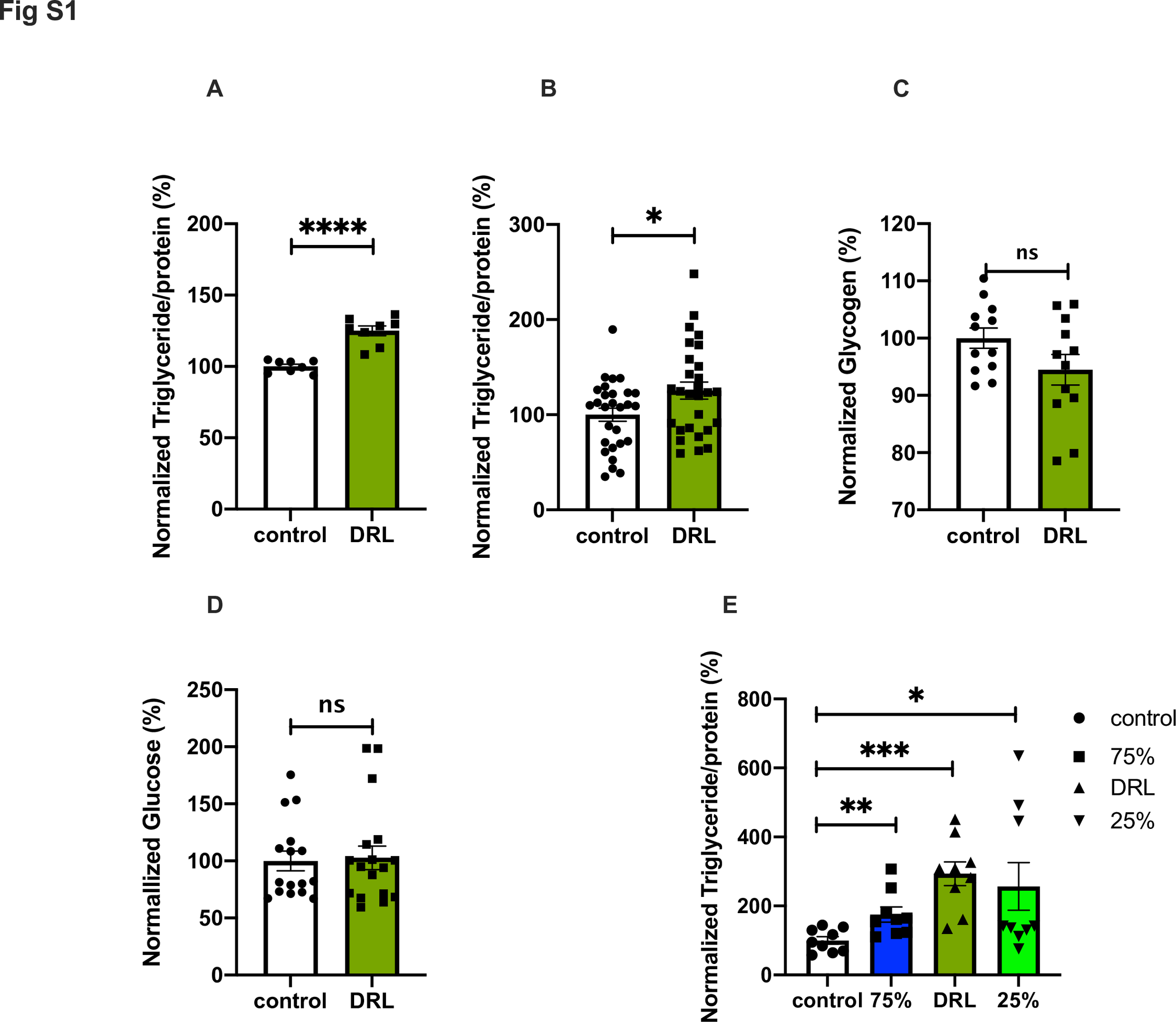

Supplement: S1 Fig — (A) Triglyceride levels are higher in DRL pre-pupae in comparison with control pre-pupae, data is shown as percentage ratio of triglyceride to total protein levels, normalised to 100% control pre-pupae [independent biological replicates = 6; p-value = 0.0001; Unpaired Students t-test with Welch Correction]. (B) Triglyceride levels are higher in DRL freshly eclosed flies when compared to control flies, data is shown as percentage ratio of triglyceride to total protein levels, normalised to 100% control flies [independent biological replicates = 28; p-value = 0.0296; Unpaired Students t-test with Welch Correction]. (C) Glycogen content remains unchanged in 5 day old DRL and control flies, data is shown as percentage glycogen content normalised to 100% control flies [independent biological replicates = 12; p-value = 0.1007; Unpaired Students t-test with Welch Correction]. (D) Glucose content shows no significant difference in 5 day old DRL and control flies, data is shown as percentage glucose level normalised to 100% control flies [control n = 16; DRL n = 18; p-value = 0.7984; Mann-Whitney test]. (E) Triglyceride levels are higher in mature flies that emerged from larvae fed with 75%, DRL and 25% diluted food, data is shown as percentage ratio of triglyceride to total protein levels, normalised to 100% control flies [independent biological replicates n = 9; p-value between control and 75% is = 0.0097, Unpaired Students t-test with Welch Correction; between control and DRL is = 0.0004 Unpaired Students t-test with Welch Correction, and between control and 25% is = 0.0315; Mann-Whitney test]. [p-value *<0.05; ** <0.01,*** <0.001, **** <0.0001, data is presented as mean ± SEM]. (TIF) [file pone.0247175.s001.tif]

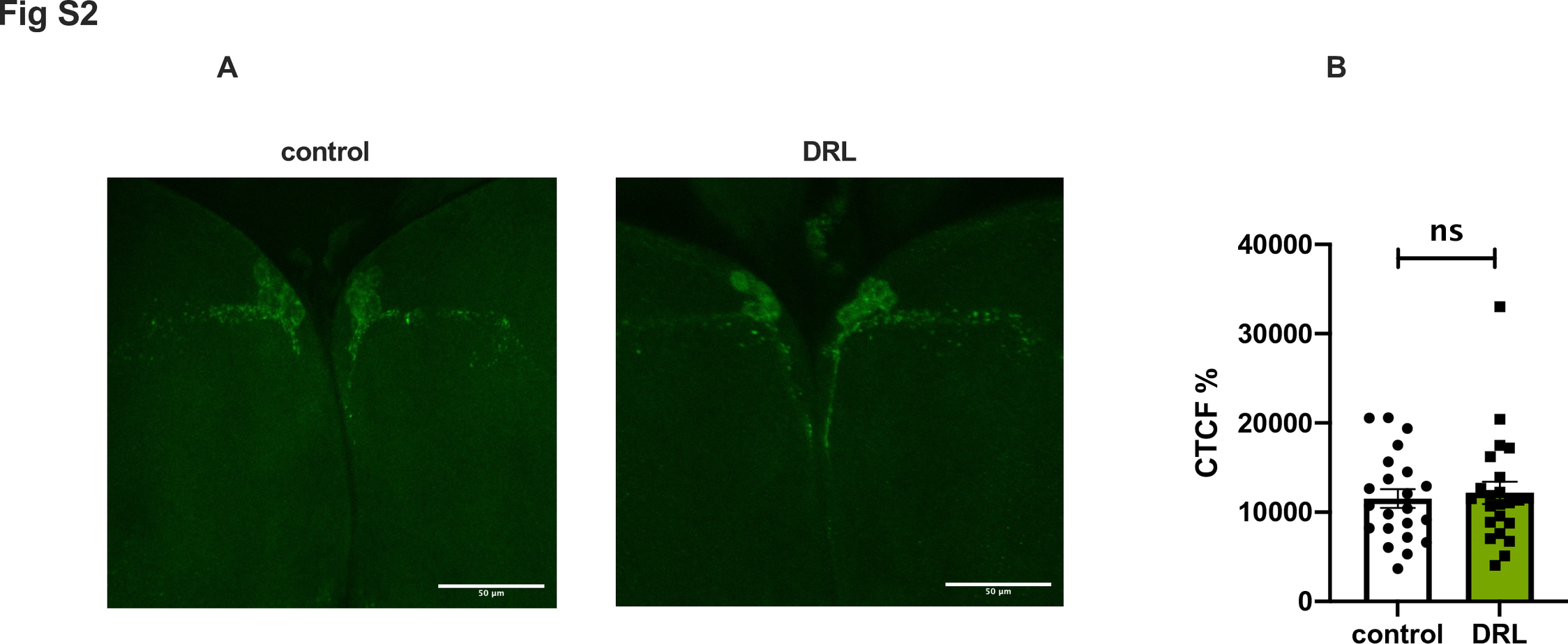

Supplement: S2 Fig — (A) DILP2 staining of IPCs in 3rd instar larvae of control and DRL. (B) Corrected Total Cell Fluorescence levels of control and DRL in IPCs, data is shown as percentage of CTCF content normalised to 100% in control larvae [independent biological replicates = 11; p-value = 0.6992; Unpaired Students t-test with Welch Correction]. [p-value *<0.05; ** <0.01,*** <0.001, **** <0.0001, data is presented as mean ± SEM]. (TIF) [file pone.0247175.s002.tif]

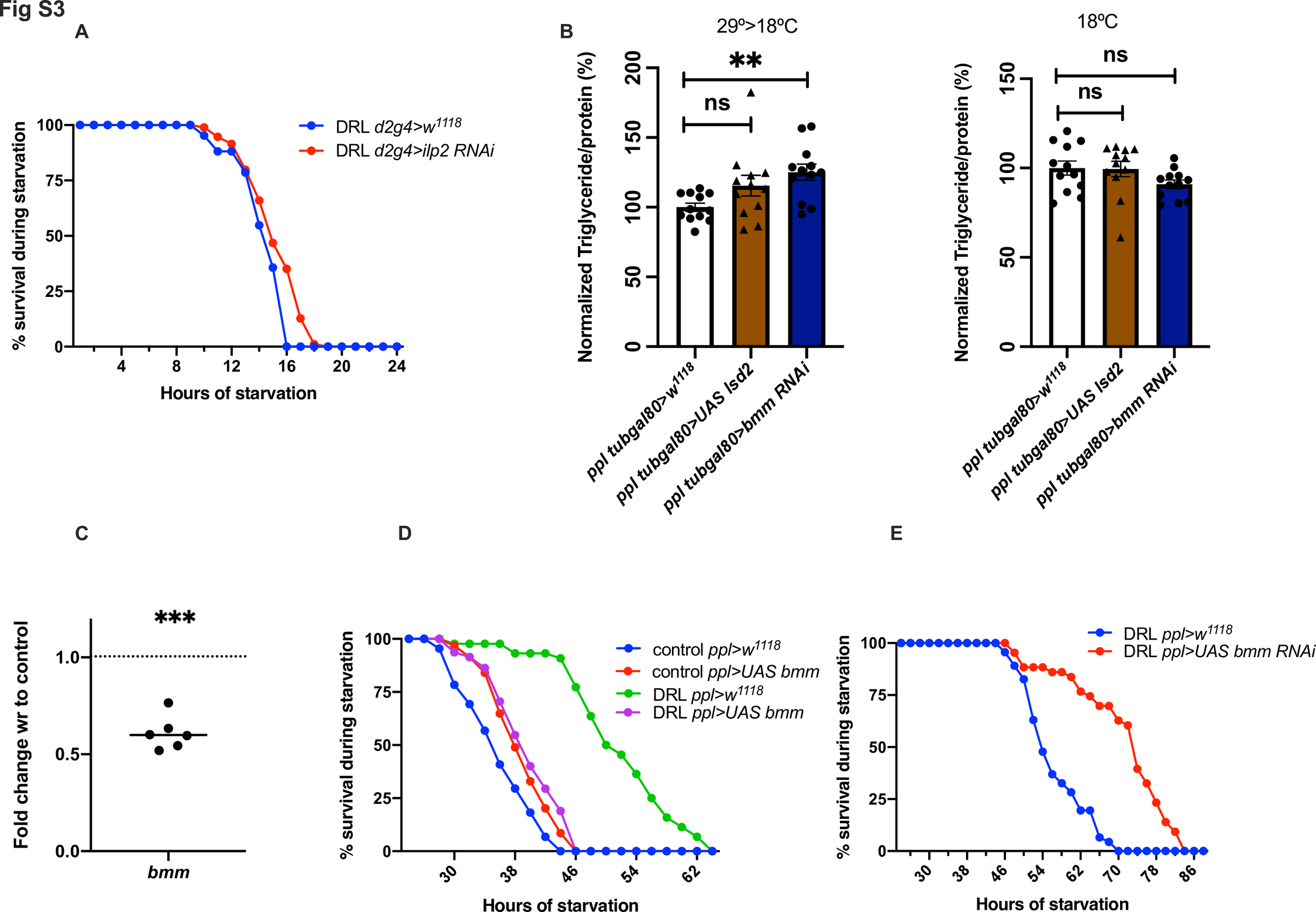

Supplement: S3 Fig — (A) Enhanced resistance to starvation in mature DRL flies is not affected by down regulation of dilp2 mRNA in the IPCs, data are shown as the percentage of DRL flies of the genotypes dilp2Gal4>w1118 and dilp2Gal4>UAS-dilp2-RNAi, which were alive at various time points of starvation, values are shown for dilp2Gal4>w1118 and dilp2Gal4>UAS-dilp2-RNAi flies fed on 50% diet during larval stages [independent biological replicates = 3, p-value between DRL dilp2Gal4>w1118 and dilp2Gal4>UAS-dilp2-RNAi flies is = 0.0011; Log-rank test]. (B) Over expression of lsd2 in the larval stages alone did not make any significant change in the fat levels in mature adults. pplGal4; tubGal80ts> w1118 and pplGal4; tubGal80ts> UAS-lsd2 normally fed during larval stages were maintained at 29°C till late larval stages and transferred to 18°C till adult stages. [independent biological replicates = 12; p-values between pplGal4; tubGal80ts> w1118 and pplGal4; tubGal80ts> UAS-lsd2 = 0.0649; Unpaired Students t-test with Welch Correction]. Control experiments for tubGal80ts were maintained at 18°C from embryo till adult stages. [independent biological replicates = 12; p-values between pplGal4; tubGal80ts> w1118 and pplGal4; tubGal80ts> UAS-lsd2 = 0.9313]. Down regulation of bmm in the larval stages alone can significantly increase the fat levels in mature adults. pplGal4; tubGal80ts> w1118 and pplGal4; tubGal80ts> UAS-bmm RNAi normally fed during larval stages were maintained at 29°C till late larval stages and transferred to 18°C till adult stages. [independent biological replicates = 12; p-values between pplGal4; tubgal80>w1118 and pplGal4; tubgal80> UAS-bmm RNAi = 0.0013; Unpaired Students t-test with Welch Correction]. Control experiments for tub-Gal80ts activity were maintained at 18°C from embryo till adult stages. [independent biological replicates = 12; p-values between pplGal4; tubgal80>w1118 and pplGal4; tubgal80> UAS-bmm RNAi = 0.0640].(C) bmm levels in control and DRL freshly eclosed f [file pone.0247175.s003.tif]
